# Solid Earth–atmosphere interaction forces during the 15 January 2022 Tonga eruption

**DOI:** 10.1126/sciadv.add4931

**Published:** 2023-01-11

**Authors:** Ricardo Garza-Girón, Thorne Lay, Frederick Pollitz, Hiroo Kanamori, Luis Rivera

**Affiliations:** ^1^ Department of Earth and Planetary Sciences, University of California, Santa Cruz, Santa Cruz, CA, USA.; ^2^ Earthquake Science Center, U.S. Geological Survey, Moffett Field, CA, USA.; ^3^ Seismological Laboratory, California Institute of Technology, Pasadena, CA, USA.; ^4^ Institut Terre et Environnement Strasbourg, Université de Strasbourg/CNRS, Strasbourg, France.

## Abstract

Rapid venting of volcanic material during the 15 January 2022 Tonga eruption generated impulsive downward reaction forces on the Earth of ~2.0 × 10^13^ N that radiated seismic waves observed throughout the planet, with ~25 s source bursts persisting for ~4.5 hours. The force time history is determined by analysis of teleseismic *P* waves and Rayleigh waves with periods approximately <50 s, providing insight into the overall volcanic eruption process. The atmospheric acoustic-gravity Lamb wave expanding from the eruption produced broadband ground motions when transiting land, along with driven and conventional tsunami waves. Atmospheric standing acoustic waves near the source produced oscillatory peak forces as large as 4 × 10^12^ N, exciting resonant solid Earth Rayleigh wave motions at frequencies of 3.7 and 4.6 mHz.

## Teaser

The 2022 Tonga eruption produced ground motions dominated by force interactions between the solid Earth and atmosphere.

## INTRODUCTION

The explosive 15 January 2022 eruption of the Hunga Tonga–Hunga Ha’apai volcano produced a plume reaching 58 km high (*1*), much higher than any prior volcanic or nuclear explosion plume observed by satellites, probably abetted by the interaction between magma and water. The degree of magma-water interaction at the ocean surface or during submarine eruption remains unclear, but a strong water vapor anomaly was injected into the middle stratosphere (*2*). The event also produced almost 590,000 lightning strikes, globally observed waves in the ionosphere and atmosphere (*3*–*9*), and worldwide sea waves, in part driven by an atmospheric acoustic-gravity wave (*10*–*13*) and global seismic waves (*6*, *14*). Ashfall and tsunami waves as high as 15 m caused extensive damage on the nearby islands of the Polynesian Kingdom of Tonga, and the tsunami caused damage on many Pacific margins. The volcanic edifice involved two islands on the rim of a submerged caldera about 6 km in diameter that had merged during prior eruptions in 2015 (*15*). Small eruptions within 4 weeks preceding the main blast had expanded the joined island area, but the connecting land bridge was submerged just hours before the main blast; only small remnants of the two islands were left after the eruption, and the caldera lost a volume of about 6.5 km^3^ (*16*).

The 2022 Tonga eruption was the largest volcanic explosion since the 1991 eruption of Mount Pinatubo, in the Philippines, and possibly the most explosive eruption since the 1883 Krakatoa eruption in Indonesia or the 1912 eruption of Novarupta, in the Katmai Cluster of the Alaska Peninsula (*6*, *14*, *17*). In addition to extensive satellite observations and tsunami recordings, seismic waves from the event were recorded by global seismic stations, providing complementary information that can quantify key aspects of the source process. When global digital seismometers began to be deployed in the 1970s, long-period ground motions provided new insights into the forces, momentum transfer, and detailed time histories of volcanic eruptions such as the 1980 landslide/eruption of Mount St. Helens (*18*–*22*) and numerous more recent large eruptions (*23*–*31*). When modest size or larger eruptions, with volcanic explosivity index of ≥4, occur at seismically and geodetically well-instrumented volcanoes, it is possible to determine detailed models of the forces acting in the Earth during the eruption process, including: depressurization of deep magma reservoirs, conduit shear drag of ascending magma or plug resistance, piston collapse within the caldera, and reaction to venting and mass loss as gas, ash, and magma jet out of the system (*21*, *25*, *27*, *32*), along with any accompanying large-scale landsliding (*18*, *33*). Such detailed source complexities can only be constrained with abundant near-source geophysical information, which is not available for the 2022 Tonga eruption (the nearest high-quality seismic station is ~755 km away from the back-arc volcanic island). However, because of the size of the eruption, effective source force systems can still be well constrained using only remote seismic wave analysis, as shown here, with both direct excitation of seismic waves by the eruptive process and ground motions produced by subsequent atmosphere–solid Earth interactions constraining valuable characteristics of the complex eruption and the coupled dynamic Earth system.

## RESULTS

**Fig. 1.**
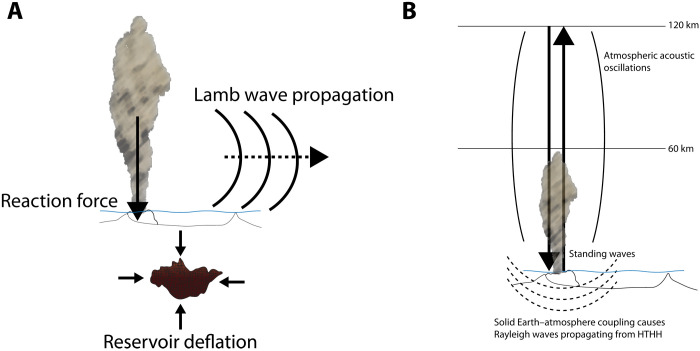
Schematic representation of the main mechanisms that contributed to ground motions during the eruption. (**A**) Downward reaction forces from jetting at the vent, which are modeled as vertical point forces, and magma reservoir deflation, which is modeled as an isotropic implosive source, produce similar waveform fits (Fig. 3 and fig. S5). The true source must be a combination of both effects. Propagation of the atmospheric acoustic-gravity pressure wave (Lamb wave) also produced ground displacements recorded at seismic stations worldwide (e.g., Fig. 5). (**B**) Atmospheric acoustic waves oscillating for ~8000 s coupled to the solid Earth, producing harmonic Rayleigh waves that radiated away from the source (Figs. 7 and (8). HTHH: Hunga Tonga-Hunga Ha'apai.

There are three fundamental contributions to ground motions detected by seismometers for the 2022 Tonga eruption: direct seismic waves from the eruptive process itself and two additional interactions associated with the atmospheric disturbances produced by the eruption. Figure 1 shows a schematic representation of these primary mechanisms, which we describe in more detail below.

The first contribution involves forces acting at the source during rapid pressure deflation within the magma reservoir and conduit system, combined with interactions of ascending material in the conduit as the system unplugs and ensuing rapid venting of material in the eruption jet (Fig. 1A). The last component of these source process forces is a direct solid Earth–atmosphere interaction and has consistently been observed to be well represented by a reaction force applied to the Earth at the vent site, with a force time history that indicates the eruption history (*14*, *18*–*20*, *25*). If the eruption was accompanied by partial collapse of the caldera, then there could also be contribution to ground displacements from high-angle normal faulting on the ring fault, and it would be almost indistinguishable from a single vertical point force. The true source will involve a complex combination of almost coincident force systems acting to lower pressure in the magmatic system, to move material to the surface, and to jet it out into the atmosphere, so overall seismic motions and any force representations found will involve some trade-off between different contributions. Past considerations of remote seismic wave contributions from implosive and reaction forces indicate that the latter dominates (*18*) and certainly must occur as the volcanic plume rises. Seismic wave amplitudes have thus been used to anticipate plume heights because of their sensitivity to the reaction force (*34*).

The other two contributions are related to atmospheric instabilities that are seldom observed in nature. One is the air pressure acoustic-gravity wave that expands cylindrically away from the source at about 0.305 km/s, which is called a Lamb wave (*7*, *8*, *35*). Passage of the high-pressure wave causes accelerations observed on the vertical components of seismometers, while pressure loading of topography causes tilting observed on the horizontal components, producing readily observable transient motions as the Lamb wave passes over land (*6*). The corresponding seismic recordings can provide an estimate of the atmospheric pressure to supplement barometric pressure measurements, and the ground tilting may be of local interest, but it is important not to confuse the motions with direct source process excitation. The second atmospheric disturbance involves acoustic oscillations excited by the eruption, which can produce periodic loading of the near-source ground, coupling to the solid Earth to produce quasi-monochromatic Rayleigh waves that spread away from the source (*35*–*40*). Acoustic coupling between the atmosphere and solid Earth may dominate the surface wave excitation (*40*).

Previous publications on the event have provided a broad range of geophysical observations that show the coupled nature of the atmosphere, the ocean, and the solid Earth but have focused most of their attention on the phenomena related to the Lamb wave (*6*–*8*), although at least one study has used seismic records to estimate an average force amplitude spectrum of the eruption (*14*). Here, we consider the solid Earth–atmosphere interactions that produce seismic waves emanating from the source to provide insights into the seismic source processes throughout the most energetic stage of the eruption. As will be shown, this analysis provides a detailed time-varying characterization of the direct volcanic source process and the rippling atmospheric effects that the eruption triggered, with precise time resolution.

### Direct reaction forces

**Fig. 2.**
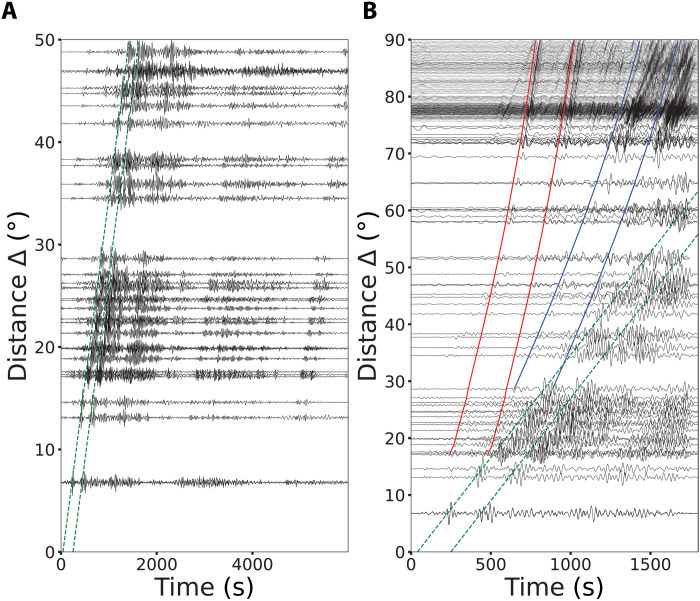
Vertical component profiles obtained from GSN, GEOSCOPE, and other broadband stations, bandpass filtered from 0.01 to 0.05 Hz, exemplifying seismic *P*, *S*, and *R*_1_ arrivals for the first two blasts. (**A**) Displacement records section showing the *R*_1_ arrival (dashed green lines) of the first two subevents *E*_1_ and *E*_2_ (Fig. 3). (**B**) Displacement records of all the stations used in this study. Solid red and blue and green dashed curves indicate the seismic wave arrivals *P*, *S*, and *R*_1_, respectively. *P* waves from these records quantify the time history of forcing from the first two volcanic subevents within 6 min of the *M*5.8 origin time. While *P* waves from later volcanic subevents cannot be identified because of interference from other phases, the relatively large-amplitude Rayleigh waves reveal numerous volcanic subevents recorded across all distances, allowing quantification of the time history of forcing out to ~4.5 hours after the main blast.

**Fig. 3.**
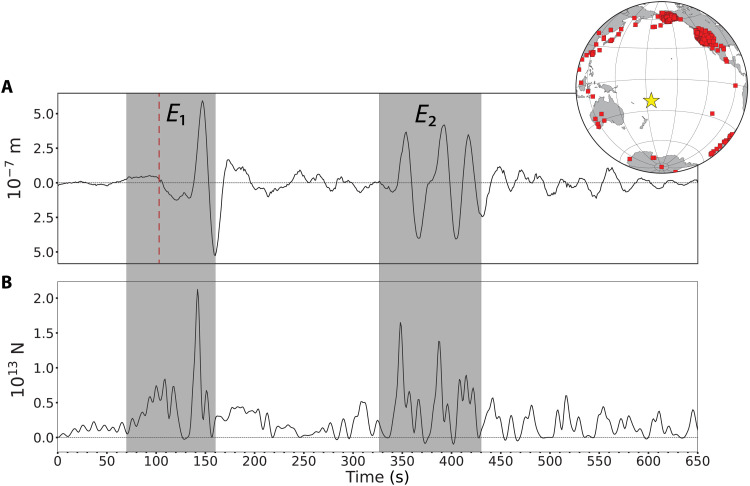
Global *P*-wave ground motion and reaction force time series. (**A**) Global stack of 518 vertical component *P*-wave ground motions from stations shown in the inset map, filtered in the passband of 0.01 to 0.05 Hz and corrected to a reference distance of 78.5°, which is the median distance of all stations used for the body wave analysis. Time is from 100 s before the *P*-wave arrival time at 78.5° [04:26:46 UTC, red dashed line] relative to the origin time of the *M*5.8 event (04:14:45 UTC), located by the USGS. Note the smooth low-amplitude long-period energy within 40 s preceding the expected arrival. (**B**) Vertical downward (reaction) point-force time history obtained by deconvolving the global stack by an impulse response Green’s function, with imposed positivity constraint. The first two main volcanic events *E*_1_ and *E*_2_ are shown in the gray boxes.

Axisymmetric radiation of body waves and surface waves from the source location is expected for rapid vertical venting during an explosive eruption (Fig. 1A). The forces acting at the source produced teleseismic *P* waves with little azimuthal waveform or amplitude variability (Fig. 2 and fig. S1), allowing the aligned signals to be stacked (averaged) to give a representative ground motion time history for the 2022 Tonga eruption (Fig. 3). The stacked *P* waveform at an epicentral distance of 78.5° (8732 km), obtained from 518 broadband ground displacement seismograms from stations at epicentral distances of 50° to 90° (5550 to 9990 km), is shown in Fig. 3A. The signal is bandpass filtered in the frequency band 0.01-0.05 Hz, with a causal Butterworth filter. The U.S. Geological Survey National Earthquake Information Center (USGS-NEIC) reported a magnitude (*M*) 5.8 event at 20.546°S, 175.390°W at 04:14:45 UTC on 15 January, based on detectable short-period ground shaking likely produced early in the eruption. A *M*4.7 earthquake in the vicinity was reported 103 s earlier. The expected arrival time of the *P* wave from the *M*5.8 event is shown by the red dashed line in Fig. 3A. The data shows a smooth low-amplitude long-period upward motion beginning about 40 s before a downward motion that begins near the theoretical arrival time of the 5.8 event, followed by a strong upward pulse with a peak amplitude of 0.56 μm. A sequence of three pulses is seen 200 s later, and there are later weaker arrivals, all evident in stacks for different passbands (fig. S2).

*P* waves are the fastest traveling seismic waves, and at the distance of the stacked waveform, the propagation effects are relatively simple, especially for a point force mechanism, so this waveform can be modeled by assuming a standard Earth velocity model and attenuation structure along with a specific source force system. Using impulse responses (Green’s functions) for different standard Earth models (Preliminary Reference Earth Model, PREM, and AK135) with various source force representations, we determine corresponding time histories of the eruptive force system by deconvolving the data by a particular Green’s function (fig. S3B). Figure 3B shows the force time history, *F*(*t*), obtained for a vertical, downward reaction force acting on the surface of the Earth as eruption venting occurs, with the deconvolution constrained to have only positive downward forces, which provides excellent waveform fitting (fig. S3D). This reveals a broad low-amplitude pulse that begins about 40 s ahead of the *M*5.8 arrival, with an abrupt drop to zero, followed by a sharp peak force with a peak value of 2.1 × 10^13^ N. Multiple peaks of somewhat lower peak force produce the set of arrivals 200 s later. Similar *F*(*t*) values are found for *P* data with different passbands, and in all cases, the waveforms are very well fit (fig. S4).

As stated above, deflation in the reservoir caused by the rapid ejection of gasses and materials could be an important contributor to ground displacements. How much can the fast depressurization influence the global observations? Could such a mechanism predict certain parts of the displacement record better than the reaction force? We address these questions by performing the deconvolution of the displacement record with an isotropic implosion moment tensor at a depth of 5 km, which is the minimum depth for the magma reservoir at Hunga Tonga–Hunga Ha’apai as reported by others (*15*). The isotropic source deconvolution can fit the whole body wave record and the point force (fig. S5); we assert that this is due to the limited data bandwidth as found for previous eruptions (*18*). Ultimately, both pressure reduction and reaction to the jetting must occur, so Fig. 3 represents the net force acting to produce the seismic motions. Details of the interfering forces cannot be resolved teleseismically, and this holds for attempts to invert for moment-tensor representations as well.

**Fig. 4.**
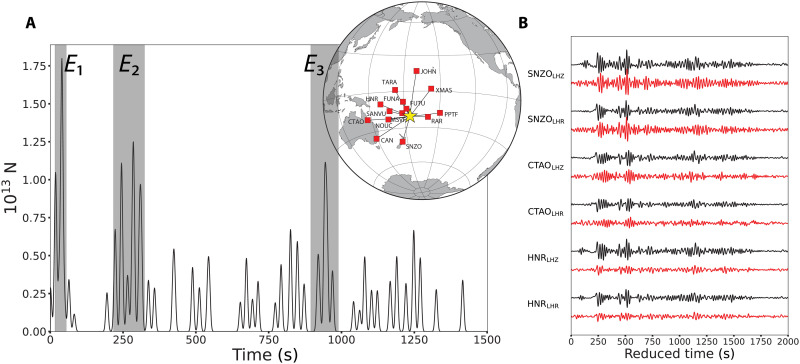
Simulated annealing inversion results. (**A**) Vertical point-force time history obtained by fitting a series of Gaussian pulses convolved with impulse responses to surface wave ground velocity records at the 14 stations shown in the inset map. Time is with respect to the *M*5.8 origin time. (**B**) Examples of the fit of synthetic displacement records from the simulated annealing inversion (red) to data (black). The first three main volcanic events *E*_1_, *E*_2_, and *E*_3_ are shown in the gray boxes. *E*_1_ and *E*_3_ were detected by infrasound stations worldwide (*7*), but *E*_2_ did not seem to be accompanied by an infrasound signal.

The *P*-wave analysis is limited to the first 10 min of the process because multiple seismic phases arrive later, but much longer time intervals can be studied by examination of surface waves at stations with epicentral distances less than 50°. The data shows two dominant packets of Rayleigh waves separated by 200 s (Fig. 2 and fig. S6), moving away from the source region, followed by later wave packets. A joint waveform inversion for signals filtered in the 0.01-0.05 Hz passband was performed for 14 azimuthally distributed stations, assuming a vertical point force and providing an *F*(*t*) with pulses persisting for more than 15,500 s (Fig. 4A and fig. S7). The maximum force strength is 1.8 × 10^13^ N. This source representation fits the dominant Rayleigh wave packets on the vertical and radial components (Fig. 4B).

**Fig. 5.**
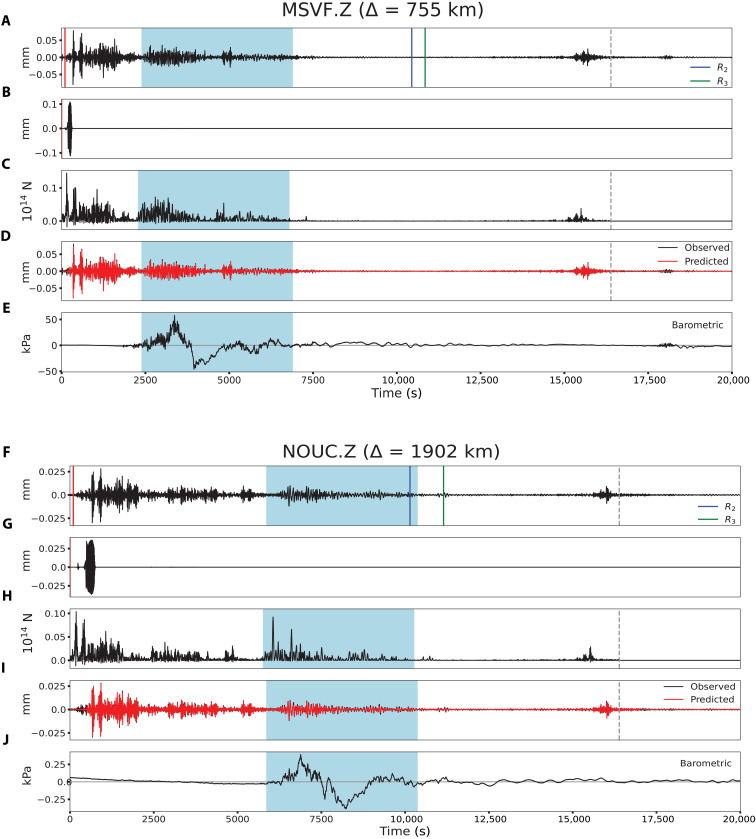
The vertical point-force deconvolutions for vertical ground motion surface waves at stations MSVF and NOUC showing atmospheric Lamb wave contamination. (**A**) The vertical component ground motion record at station MSVF, at 755 km from the source, filtered in the passband of 0.01 to 0.1 Hz. The red line indicates the origin time of the 5.8 event reported by the USGS-NEIC. The blue and green lines correspond to the arrival times of *R*_2_ (long arc) and *R*_3_ (second passage of *R*_1_) Rayleigh wave phases, respectively. (**B**) Impulse response synthetic seismogram for a downward vertical point force at the surface of model AK135 with a strength of 10^14^ N. (**C**) The force time history, *F*(*t*), obtained by positivity-constrained iterative deconvolution shows a ~16,500-s-long history of the eruptive process. (**D**) Observed (black) and reconstituted (red) ground motions obtained from convolution of the impulse response (B) and *F*(*t*) (C). (**E**) Barometric pressure recording at MSVF showing the atmospheric Lamb wave arrival during the blue shaded interval, which produced contamination of ground motion and the estimated *F*(*t*) in the corresponding arrival window (blue shaded area), seen in (A), (C), and (D). (**F**) The vertical component ground motion record at station NOUC, at 1902 km from the source, filtered in the passband of 0.01 to 0.1 Hz. (**G**) Impulse response synthetic seismogram for a downward vertical point force at the surface of model AK135 with a strength of 10^14^ N. (**H**) The force time history, *F*(*t*), obtained by positivity-constrained iterative deconvolution. (**I**) Observed (black) and reconstituted (red) ground motions obtained from convolution of the impulse response (G) and *F*(*t*) (H). (**J**) Barometric pressure recording at NOUC. The Lamb wave arrives at different times for stations at different distances, so by masking out the corresponding window for each station, the composite stack can recover uncontaminated *F*(*t*) for the entire time range.

**Fig. 6.**
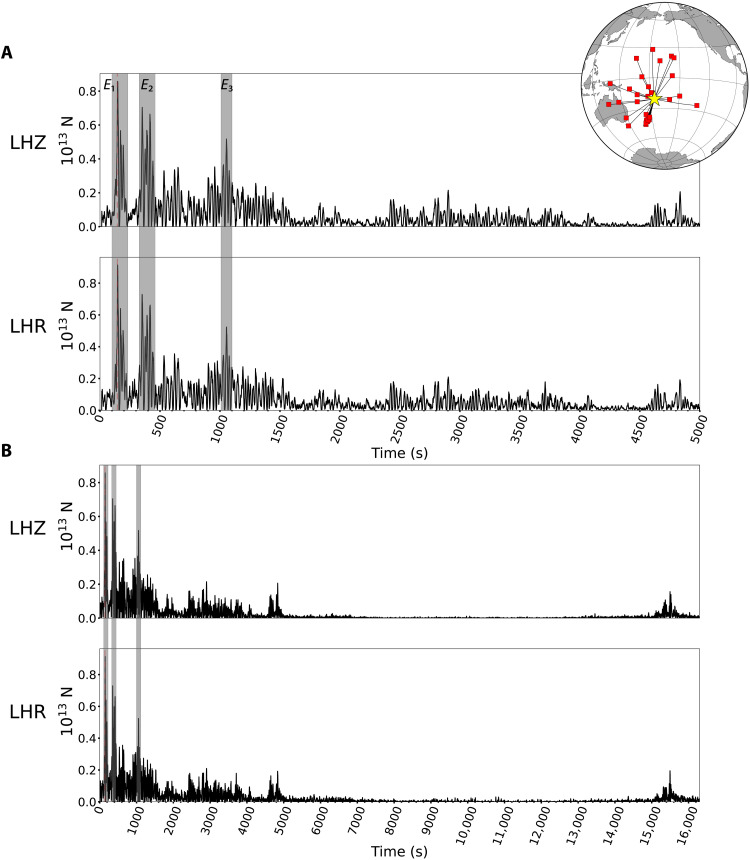
Vertical reaction force time history from Rayleigh waves. The median stack vertical point-force time histories obtained by deconvolving individual displacement recordings extending for 5000 (**A**) and 16,300 s (**B**) after the source began by corresponding Green’s functions for vertical motion (LHZ) and horizontal motion (LHR) of short-arc Rayleigh waves. The inset map shows locations of the 30 stations used. Consistency of the LHZ and LHR time series confirms that the motions are Rayleigh waves. Intervals of the seismograms when ground motion was contaminated by the passage of the atmospheric Lamb pulse (Fig. 5) were screened out before stacking the individual force time histories. The first three main volcanic events *E*_1_, *E*_2_, and *E*_3_ are shown in the gray boxes.

Deconvolution of individual vertical and radial Rayleigh wave components using vertical point-force impulse responses for model AK135 also provides estimates of *F*(*t*) station by station. Figure 5 shows examples of the surface wave deconvolution results for the whole ~4.5 hours of eruptive sequence at stations MSVF and NOUC. The effect of the propagating Lamb pulse on the ground motions is evident, and it is marked by a shaded blue area. The median stacks of 27 station deconvolutions in the 0.01-0.1 Hz passband for the first 5000 s of the eruption are shown in Fig. 6A and for the 4.5-hour duration in Fig. 6B. Very similar *F*(*t*) are resolved for the two components, demonstrating that these are truly Rayleigh waves from the source. In stacking the *F*(*t*), we suppressed intervals when passage of the Lamb pulse perturbed the ground motion recordings. The *F*(*t*) estimated from Rayleigh wave inversion and deconvolution are similar to that from *P* waves for the first 600 s, with two main bursts of pulses (*E*_1_ and *E*_2_) 200 s apart. Precise features differ between methods because of differences in dispersion predictions relative to the data for surface waves (figs. S8 to S10). However, a comparison between the *F*(*t*) estimated by surface wave deconvolution using the AK135 Earth model and the *F*(*t*) computed by the joint waveform inversion shows great similarity between the two, despite the former having double the amplitudes of the latter (fig. S11), strengthening the robustness of our results. In addition to clearly identifying the main bursts of activity *E*_1_, *E*_2_, and *E*_3_ in the deconvolution and inversion results, there are other signals that also appear in both. For example, the signals between *E*_2_ and *E*_3_, which have peak amplitudes of approximately half the largest signal, are present in the inversion results (Fig. 4) as well as in the deconvolution results using the two Earth models (Fig. 6 and figs. S9 and S10). We interpret these signals as representing source energy from the eruptive process. Similarly, the signals between 15,000 and 16,000 s (Fig. 6 and fig. S7) are observed in both results. These signals produced infrasound and hydroacoustic phases (*8*, *41*) and also match the timing of volcanic activity as observed in satellite imagery (*42*), suggesting that this was a secondary explosive stage of the 15 January 2022 eruptive period. The events identified as *E*_1_ and *E*_3_ are associated with widely recorded infrasound events (*8*, *41*), whereas *E*_2_ is not. We believe that the reason *E*_2_ is not associated with an infrasound arrival is that it only began ~200 s after *E*_1_, making it difficult to isolate in the dispersed infrasound signal and coda of the earlier event, whereas our high-resolution seismic data deconvolution and inversion clearly resolve the signals in *E*_2_. Furthermore, there is evidence of *E*_2_ producing an increase in signal-to-noise ratio at hydroacoustic stations around the world (*41*).

The *P*-wave and Rayleigh wave signals demonstrate the sequence of explosive eruptions lasting for about 4.5 hours that generated reaction forces on the Earth. This characterizes the overall direct seismic response to the eruption process, dominated by venting reaction forces but including destructive interference from pressure reduction, conduit drag, and possible caldera collapse.

### Resonant forces

**Fig. 7.**
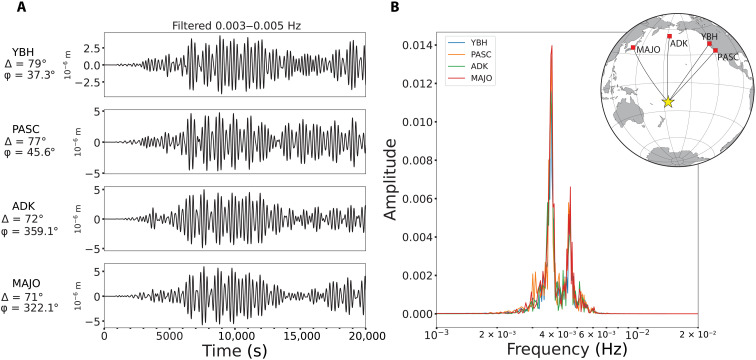
Long-period Rayleigh waves generated by resonating atmospheric acoustic modes coupled with the solid Earth at the source region. (**A**) Ground displacements, filtered in the 0.003- to 0.005-Hz passband, recorded at stations ADK, MAJO, PASC, and YBH at varying epicentral distances (Δ) and azimuths from the source (ϕ). (**B**) Spectra of the vertical ground motions filtered in the 0.003- to 0.005-Hz passband. Spectral peaks at 3.4 and 4.6 mHz correspond to the frequencies of atmospheric acoustic standing wave modes that couple to the ground to excite Rayleigh waves that spread from the source region.

**Fig. 8.**
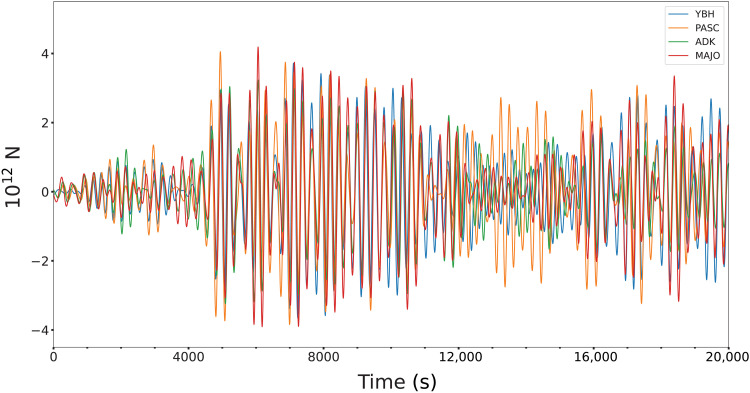
Oscillatory force time history of standing acoustic waves obtained by deconvolving the records with an impulse response for a single vertical point force, without positivity constraint. Time is relative to the USGS-NEIC *M*5.8 event.

Narrow-band long-period ground motions for the 2022 Tonga eruption show resonant Rayleigh waves similar to those observed for the 1991 Pinatubo eruption (*35*–*40*) at many global stations. Example signals in the passband from 3 to 5 mHz are shown in Fig. 7A, with modulating packets of motion that correspond to interference of two monochromatic traveling waves with dominant frequencies of 3.7 (270 s) and 4.6 mHz (217 s) (Fig. 7B). The former is identified as an atmospheric acoustic mode with energy concentrated between the mesosphere and the ground and trapped both vertically and horizontally (Fig. 1B) (*40*). Efficient coupling of acoustic energy may account for the greater strength of the 3.7-mHz excitation (*40*). The reverberations are established by about 3000 s after the origin and involve at least 8000 s of oscillatory forces applied by the atmosphere on the Earth’s surface exciting the discrete frequencies of Rayleigh waves. The time window for reliable detection is constrained by arrivals of long-arc great-circle Rayleigh waves (*R*_2_), which overlap any direct waves after about 9000 s. Similar signals have been interpreted for the 1991 Pinatubo eruption as loading from acoustic standing waves reverberating within the atmosphere coupling to the solid Earth. Deconvolution by a time-varying vertical force that can alternate in sign, reflecting the local harmonic motion of the air column, provides the individual *F*(*t*) functions shown in Fig. 8 with peak strengths of ~4 × 10^12^ N. The oscillatory forces acting at the site of the source show a large amplification at around 4500 s after the *M*5.8 event, indicating the time that it takes for the atmospheric acoustic standing modes to develop.

## DISCUSSION

Our results have profound implications for the dynamics of rare very-large volcanic eruptions. The 2022 Tonga eruption activated wave motions observed in the land, sea, and atmosphere. Here, we have focused on the propagating elastic waves excited in the solid Earth to characterize the forces acting at the source during ~4.5 hours of the most energetic stage of the eruptive process with the best time resolution available. The bursty nature of the eruption time history with many ~25 s pulses indicates a self-throttling process of the venting, possibly associated with magma fragmentation due to the interaction between water and melt (*43*, *44*) or dynamic changes in conduit stability (*45*).

The time-integrated cumulative force (momentum transfer) acting on the Earth during many pulses over these 16,000 s of the eruption was ~3.0 × 10^15^ N-s (fig. S12), which is about a factor of 2 larger than a value previously estimated using the overall amplitude spectrum for the entire process (*14*). The estimation of this force impulse strongly depends on the positivity constraint of the force time function; thus, its uncertainty increases ~10 min after the origin time of the *M*5.8 event, since the atmospheric oscillations are well established after that time, and we cannot guarantee that the forces acting on the solid Earth are purely positive. The peak force strengths for the eruption are about an order of magnitude larger than for the 1980 Mount St. Helens eruption (*19*, *20*). Given that there is not an analogous study performed for the 1991 Pinatubo eruption, whether the Hunga Tonga–Hunga Ha’apai eruption was the most energetic volcanic event in a century remains unclear. Our seismological force impulse model implies equal momentum transfer into the Earth’s atmosphere, so that estimates of driving forces from atmosphere dynamics and seismic wave propagation can be compared for consistency. Currently, on the basis of satellite observations of the expanding plume combined with a one-dimensional gravity-driven cloud spreading model (*46*), the estimated average mass discharge rate over the first 100 min of the sequence is 3.3 × 10^9^ kg/s (*47*), which is on the same order of magnitude as the mass discharge rate calculated for the 1980 Mount St. Helens and 1991 Pinatubo eruptions (*21*, *48*) and one order of magnitude larger than the 1982 El Chichón eruption (*49*). A nominal discharge rate of 300 m/s (at about the atmospheric sound velocity) then suggests an average force amplitude of ~10^12^ N, roughly consistent with the average force amplitude from our seismological estimates. Further interactions between the land and air were sustained by atmospheric acoustic oscillations exciting over 8000 s of narrow-band Rayleigh waves spreading from the source.

## MATERIALS AND METHODS

### Materials

#### Seismic data and barometric pressure data

All waveform data were downloaded using the Wilber 3 server (https://ds.iris.edu/wilber3/) of the Incorporated Research Institutions for Seismology (IRIS). For the body wave analysis, we obtained long-period (LH) and broadband (BH) data for all available networks with stations from 50° to 90° from the event hypocenter reported by the USGS-NEIC (latitude = −20.546, longitude = −175.390, and depth = 0 km). For the surface wave analysis, we obtained long-period (LH) seismic data for all stations within 50° from the USGS-NEIC source location. Similarly, for analyzing the effect of resonant atmospheric standing wave forces at the source, we requested long-period (LH) data for stations at distances between 70° and 80°. The barometric pressure data (LD) were accessed from the IRIS server through the Python package ObsPy (*50*).

#### Green’s functions and models

The theoretical Green’s functions (TGFs) used in deconvolutions were obtained from the IRIS Synthetic Engine (Syngine) (IRIS: Data Services Products: Syngine), which provides a database of precomputed synthetic seismograms for various types of point sources and different Earth models. To model the source process as the reaction force from the eruption jet, we extracted TGFs for a downward vertical point force of 10^14^ N (as a reference force) acting at the surface at the coordinates of the USGS-NEIC hypocenter mentioned above. To model the source contribution from pressure reduction in the magma reservoir, we extracted TGFs for isotropic implosive moment tensors with a scalar moment of 10^15^ Nm (again, as a reference source strength) for depths of 1 to 12 km. For the vertical point-force TGFs and the implosive source TGFs, we used a Gaussian source duration of 4 s, which is the shortest period impulse response available on Syngine. To assess the effect of the Earth model on our results, we used TGFs for the AK135 (*51*) and PREM_a (anisotropic) (*52*) one-dimensional velocity, density, and attenuation models.

For the *P*-wave analysis, because the propagation of the wavefield is axisymmetric for a vertical point force, we only used the TGFs at the median distance to all the stations (78.5°), for which we stacked the amplitude-normalized displacement pulses and scaled the peak amplitude by the median distance–corrected peak amplitude. For the surface wave analysis, we requested TGFs for each station to account for dispersion differences.

### Methods

To determine the effective teleseismic force time history *F*(*t*) at the eruptive source, we deconvolved TGFs from the stacked or individual ground displacement records by performing iterative time-domain deconvolutions (*53*, *54*), or we directly invert the displacement waveform recordings. The deconvolution procedure is constrained to construct a positive *F*(*t*), iteratively fitting the observations by a sequence of scaled and shifted impulse responses in a least-squares sense.

#### Instrument deconvolution specifics

The original recorded seismograms were demeaned and detrended and then deconvolved by the corresponding instrument response over a very broadband range (0.001-0.5 Hz for LH and 0.001-10.0 Hz for BH) by frequency domain deconvolution and/or recursive deconvolution. The resulting broadband ground displacements were then bandpass filtered with Butterworth bandpass filters with designated low- and high-frequency corners, using a single-pass (causal) filter (for *P* waves) that preserves first-arrival information with no precursory side lobes or a two-pass (acausal) filter (for Rayleigh waves). Passbands of 0.01-0.05, 0.01-0.1, 0.01-0.2, and 0.01-0.4 Hz were used for *P*-wave analysis and 0.01-0.05 and 0.01-0.1 Hz for surface wave modeling and deconvolution, respectively. For long-period Rayleigh wave analysis, a passband of 0.003-0.005 Hz was used. We downsampled all BH data to 1 Hz (the same sampling rate of the LH data) and applied a Hanning taper of 5% to each end of the bandpassed ground displacement time series.

#### P-wave data amplitudes, weighted stacking, and various passbands

The most important propagation effects for teleseismic body waves from a surface source at epicentral distances of 50° to 90° from a source are geometric spread and attenuation. The propagation effects primarily depend on the velocity structure, and model dependence can be addressed by using different Earth models with specific isotropic or anisotropic velocity, density, and attenuation profiles. For geometric spreading of the *P* waves, we corrected the amplitude of the ground displacement peak at each station by equalizing the amplitude to a median distance of 78.5° using the Jeffreys-Bullen Earth model (*55*) and a near-source velocity of 5.5 km/s.

The bandpass-filtered vertical component *P*-wave ground displacements were screened for good signal-to-noise ratio near the predicted *P* arrival time, and 518 recordings were retained. The *P*-wave stack was obtained by first prestacking all retained waveforms recorded from 50° to 90° in 20° azimuthal bins with amplitudes normalized by their peak values and then stacking the binned stacks to give a global stack. For each stack, we align the traces by calculating maximum cross-correlation lag times for windows starting 60 s before the theoretical arrival time for the USGS-NEIC *M*5.8 event and ending 150 s after that predicted arrival time. Median stacks are computed to reduce the influence of noise in the retained data. We scale the final normalized median stack by the median value of the equalized amplitudes for all data and perform a final shift relative to the theoretical arrival at the median distance.

To assess the effect of filtering the data in different passbands for the procedure described above, we perform the same steps for vertical component *P* waveforms filtered in four different frequency bands: 0.01-0.05, 0.01-0.1, 0.01-0.2, and 0.01-0.4 Hz. Figure S2 shows that, for all passbands, we observe small-amplitude positive displacements starting at ~700 s (about 50 s ahead of the expected arrival for the USGS-NEIC origin time), followed by a ~40-s-wide negative ground motion that commences close to the expected arrival time, and then a peak positive displacement with a pulse width of about 30 s. The equalized median amplitude is similar for the global stacks with different passband filters. The use of a causal bandpass filter produces overshoot of the signals, so negative amplitudes will follow positive motions.

#### Rayleigh wave simulated annealing inversion

To invert for a force time history using regional seismic data, seismic waveforms were assembled from three-component broadband records of 14 stations of the Global Seismic Network and the French GEOSCOPE Network within 4500 km of the epicenter. These were bandpass filtered from 0.01 to 0.05 Hz and corrected to velocity. Green’s functions for the impulse response to a vertical force were computed on isotropic PREM using the direct radial integration method (*56*). These were converted to Green’s functions for a unit Gaussian source time function with a half-width of 4 s; results are practically insensitive to the choice of half-width. Before source inversion, the three-component recordings and Green’s functions were resolved onto radial, transverse, and vertical components. Simulated annealing with a sparsity regularization (*57*) was then used to determine the source time function of vertical force excitation with components of all records assigned equal weight. We aligned traces manually by shifting records by amounts consistent with regional surface wave group velocity maps (*58*, *59*) to maximize the Rayleigh waveform fits. These time shifts are not revised in the simulated annealing procedure. Vertical forcing produces only Rayleigh waves on a spherically symmetric structure, and only the radial and vertical components are involved, so that possible noise sources on the transverse component do not influence the source time function determination. The objective function is a sum of the vertical and radial component waveform squared misfit and a weighted regularization term defined by the summed *p*th power of the impulse amplitudes. Results are stable for a range of regularization weights and *P* values between 0.3 and 1; we used *P* = 0.5 for the final results. The search for nontrivial forcing extends to 16,000 s after the USGS-NEIC origin time; events are detected within narrower windows spanning 0 to 1500, 4400 to 5000, and 15,000 to 15,700 s (fig. S7).

#### Rayleigh wave deconvolution

The teleseismic *P*-wave analysis provides a reliable estimation of *F*(*t*) for about 700 s, before the arrivals of secondary phases such as surface reflections *PP* and *PPP* complicate the signals and make it more difficult to isolate the source process. Long-period surface waves radiating from the source provide an opportunity to determine *F*(*t*) over much longer intervals of time to constrain the entire eruptive process.

The teleseismic *P*-wave observations have azimuthally distributed waveforms with pronounced similarity, allowing a global data stack and deconvolution by TGFs at a median distance. Due to dispersion, the surface waves show waveform variations for stations located at different distances from the source and at different azimuths for a given distance. The azimuthal variations are likely due to differences in crustal and upper mantle structure traversed by the waves as they propagate from the back-arc where Hunga Tonga–Hunga Ha’apai is located across lithospheric structures of varying age to islands and continental locations with seismic sensors. For the surface wave analysis, deconvolution was performed for the vertical and radial (horizontal, along a great-circle path) components of each station, effectively estimating the force time history from Rayleigh waves at different azimuths and distances. We use the ground displacement recordings bandpass filtered in the 0.01-0.1Hz frequency range with acausal Butterworth filters applied to both the data and TGFs.

#### Rayleigh wave F(t) stacking and Lamb pulse contamination suppression in stacks

To avoid contamination of the Lamb wave–perturbed ground motions on the combined *F*(*t*) from 27 stations at different distances (e.g., Fig. 5), we applied a mask to zero out 4500-s-long windows of the perturbed ground motion during the passage of the Lamb wave using a conservative fastest velocity of 0.33 km/s (blue shaded area in Fig. 5).

After calculating the force time history at each station, we aligned and stacked the *F*(*t*) traces. The traces were aligned using the maximum cross-correlation lag time for time windows that spanned the first 700 s of the traces. We computed a median stack of the shifted traces and shifted all traces relative to the stack using a 500-s time window and lastly repeated the process using a 250-s window. All traces are normalized relative to the maximum value in the 250-s window, and the final stack was obtained by calculating the nonzero median value for each sample bin. Last, to have the final median stack aligned in absolute time, we do a cross-correlation lag-time shift relative to a reference station MSVF at close distance to the source.
