# Supplementary material for: Solid Earth–atmosphere interaction forces during the 15 January 2022 Tonga eruption
Source: Sci Adv. 2023 Jan 11;9(2):eadd4931. doi: 10.1126/sciadv.add4931 (PMC9833673; doi:10.1126/sciadv.add4931)
Supplement: Supplementary file 1 — Supplementary Text Figs. S1 to S12 [file sciadv.add4931_sm.pdf]

Science Advances
AAAS

Supplementary Materials for

Solid Earth–atmosphere interaction forces during the 15 January 2022 Tonga eruption

Ricardo Garza-Girón *et al.*

Corresponding author: Ricardo Garza-Girón, rgarzagi@ucsc.edu

Sci. Adv. **9**, eadd4931 (2023)
DOI: 10.1126/sciadv.add4931

This PDF file includes:

Supplementary Text
Figs. S1 to S12

Supplementary Text

1) P wave deconvolutions for models AK135, PREM_a for vertical point-force for various passbands, and for an implosion

To assess the robustness of the P wave deconvolutions, we deconvolved the global P wave median stacks in the 4 different passbands shown in Fig. S2 with downward vertical point-force impulse responses for two different Earth models: AK135 and PREM_a. Deconvolutions for the 0.01-0.05 Hz passband performed using TGFs for both models produce good fits to the data and very similar F(t) (Fig. S3). The deconvolved force time-histories for different P wave passbands all show common peak forces at the same time, with slight variations in amplitude (Fig. S4). This indicates that there is no contamination by other high frequency phenomena (e.g., earthquakes) in either the P wave global stacks or in the deconvolved F(t).

The teleseismic data do not provide sufficient resolution to resolve the effects of different force systems that may be acting at the source. However, the smooth long-period ground motions preceding the sharp peak displacement suggest that there could be distinct or interfering source mechanisms dominating prior to the onset of the primary reaction force due to rapid mass discharge. One of the mechanisms that surely occurs prior and during the large-scale discharge of volcanic material out of the system is depressurization of the magma reservoir. We tested the

hypothesis that an isotropic implosive source at 5 km depth could account for some of the signal in the displacement waveforms by deconvolving corresponding TGFs with a scalar moment of 10^{15} Nm from the data, again using a positivity constraint. The F(t) obtained by assuming an exclusively implosive source can account for initial long-period positive pulse in the P wave arrival for the 0.01-0.05 Hz passband adequately, and it fits the overall signal as well as the single force deconvolution (Fig. S3). The deconvolved moment rate function for the implosive model has general similarity to the point-force time history. This similarity is not unexpected, given the simple shape of the impulse response functions for the two force systems (Fig. S3b, Fig. S5B), which are essentially flipped in sign. This similarity of the impulse responses leads to ambiguity in the source force system representation, and the deconvolved time series should be viewed as simplified composite time histories of the interfering combination of implosive and reaction forces that generated the P waves.

2) <u>Discussion of tangential component SH waves and Love waves</u>

Departures from axisymmetry in the source force system will result in radiation of transverse component SH waves and Love waves. For example, a non-vertical point-force will excite azimuthally varying SH waves with a two-lobed radiation pattern. We examined this possibility by analyzing rotated SH components in the body and surface wave signals. The SH waveforms for most traces had low signal-to-noise ratios (SNR). Traces with good SNR had maximum SH displacements at the arrival time predicted for the USGS-NEIC magnitude 5.8 origin time of $<5x10^{-7}$ m, but the data do not robustly resolve a two-lobed radiation pattern. For many stations, SH component ground motion was observed up to 70 s ahead of the expected arrival time, indicating an earlier source. Some of the energy ahead of the theoretical arrival could correspond to signals from a magnitude 4.7 event detected by the USGS-NEIC that occurred 103 s before the magnitude 5.8 origin time. The low SH signal amplitudes and lack of coherent behavior supports assumption of a vertical force model as the primary characterization of the source process for the body waves.

We examined all transverse component recordings for long period Love wave motions as well. Again, transverse component motions are apparent, with amplitudes that are up to about 75% as large as the Rayleigh wave signals. The Love wave observations can be accounted for by up to 30° deflection of the point-force from vertical, directed toward the WNW, but the dominant period range of the observations is from 10 to 50 s, so deflections from the great circle path and similarity of the group velocities for these periods may cause contamination of the putative transverse components. Modest deflection of a point-force from the vertical produces minor azimuthal pattern in the P and Rayleigh wave radiation, and the amplitude data for those phases do not confirm a geometry that accounts for the tangential motions, so we focus on axisymmetric vertical point force and implosive sources for the source representation.

3) <u>Model dependence of F(t): AK135, PREM_a</u>

We explored the robustness of our surface waves results by removing the point force impulse response for two different standard Earth models, AK135 and PREM_a. The latter model considers an anisotropic Earth structure and has slightly sharper changes of velocity than AK135 in the first 600 km. Fig. S8 shows examples of vertical component (Rayleigh wave) impulse responses for AK135 and PREM_a and corresponding signals filtered in the 0.01-0.05 Hz passband (used for the deconvolutions) and the 0.01-0.03 Hz passband. In the filtered passbands

the strong differences in short-period Rayleigh wave dispersion for the two models are greatly reduced, however, there are still minor differences in the Green's function waveforms, which account for the differences in the deconvolutions. The main effect is that inaccuracies in the predicted dispersion across the dominant passband of the data from 0.025-0.05 Hz result in sidelobes to primary pulses associated with the well-predicted source radiation. The sidelobes can be skewed early or late depending on the trend in dispersion discrepancy.

The median stacks for the AK135 and PREM_a models, not shifted to absolute time, are shown in Fig. S9 and Fig. S10, respectively. The comparison of ~16,500 s long F(t) shows that we can confidently resolve broad features of the eruption using the two models (Figs. S9A, S10A). The first stage of the eruption is composed of a series of force pulses that decay in amplitude with time lasting ~5000 s. The most vigorous reaction forces occurred during the first ~700 s. A secondary, and much less energetic, stage occurred at ~15,000 s and it sustained for ~1,000 s, making the total time of the eruption process ~16,000 s long.

Finer scale details of the first 700 s of the eruption (Fig. S9B and Fig. S10B) show that there are some differences in the stacked F(t) for the two Earth models. The Rayleigh wave energy is relatively narrow band, with most power in the period band 20 to 40 s. This relatively short-period band is strongly influenced by the crustal and uppermost mantle structure and the dispersion curves at these periods differ for models AK135 and PREM_a. No one-dimensional Earth model is expected to predict the dispersion accurately for the various paths sampled by the data, and errors in predicted dispersion in the impulse responses for the narrowband data will lead to side-lobes in the deconvolutions. This appears to cause some early artifacts in the PREM_a deconvolutions and likely some later artifacts in the AK135 deconvolutions, relative to common main pulses. Nevertheless, the deconvolutions for the two models show the same main characteristics of the force time history as obtained from the P waves deconvolution for the early part of the eruption. The peak force is followed by a long-period pulse, which is divided into two different pulses in the AK135 surface wave stack. The second set of three force pulses ~100s after the long-period pulse observed in the P wave deconvolution can also be seen in the two surface waves stacks, but the PREM_a F(t) seems to have some early side lobes. The detailed velocity structure of the real Earth is not known sufficiently to account for the short-period surface wave dispersion precisely, so the estimates of F(t) complexity are not uniquely resolved. The very different structures of AK135 and PREM_a likely span the range of structural mismatches expected for 1D models, so we have confidence in the common features of the deconvolutions. It appears that the main characteristics of the force system acting on the volcano as the eruption develops are captured quite well by the long surface wavetrains radiated from the source.

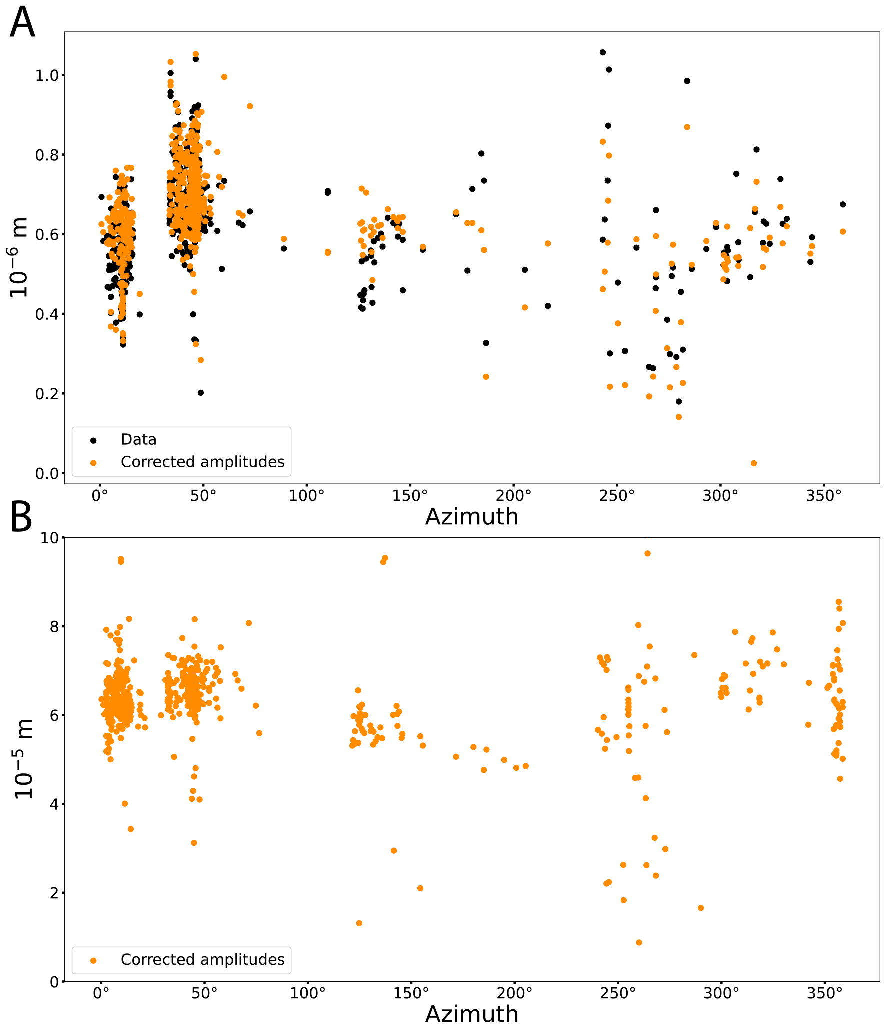

Fig. S1. – Corrected P amplitudes for the first large positive ground displacement pulse versus azimuth. A. Peak amplitudes of the instrument-corrected ground displacement signals of the January 15, 2022 Tonga eruption in the passband 0.01-0.05 Hz for the first large P wave arrival at global broadband seismic stations in the epicentral distance range 50° to 90° (black dots), with corrections for geometric spreading to a distance of 78.5° (orange dots), show little azimuthal pattern. The median values of the observations for different passbands are used to scale the normalized median stacks of bin-averaged P wave ground displacements (Fig. S2) giving the true-amplitude median ground motion stacks shown in Figs. S3 and S4. **B.** Peak amplitudes of the instrument-corrected ground displacement signals of the November 11, 2022 M_W 7.3 earthquake in the Tonga Islands in the passband 0.01-0.05 Hz for the first large P wave arrival at global broadband seismic stations in the epicentral distance range 50° to 90°, with corrections for geometric spreading and radiation pattern to a distance of 78.3°. The radiation pattern and geometric spread corrected amplitudes for the M_W 7.3 earthquake also show little azimuthal variation, with similarities with the eruption data indicating that the residual pattern is primarily due to attenuation and receiver effects and not to source effects.

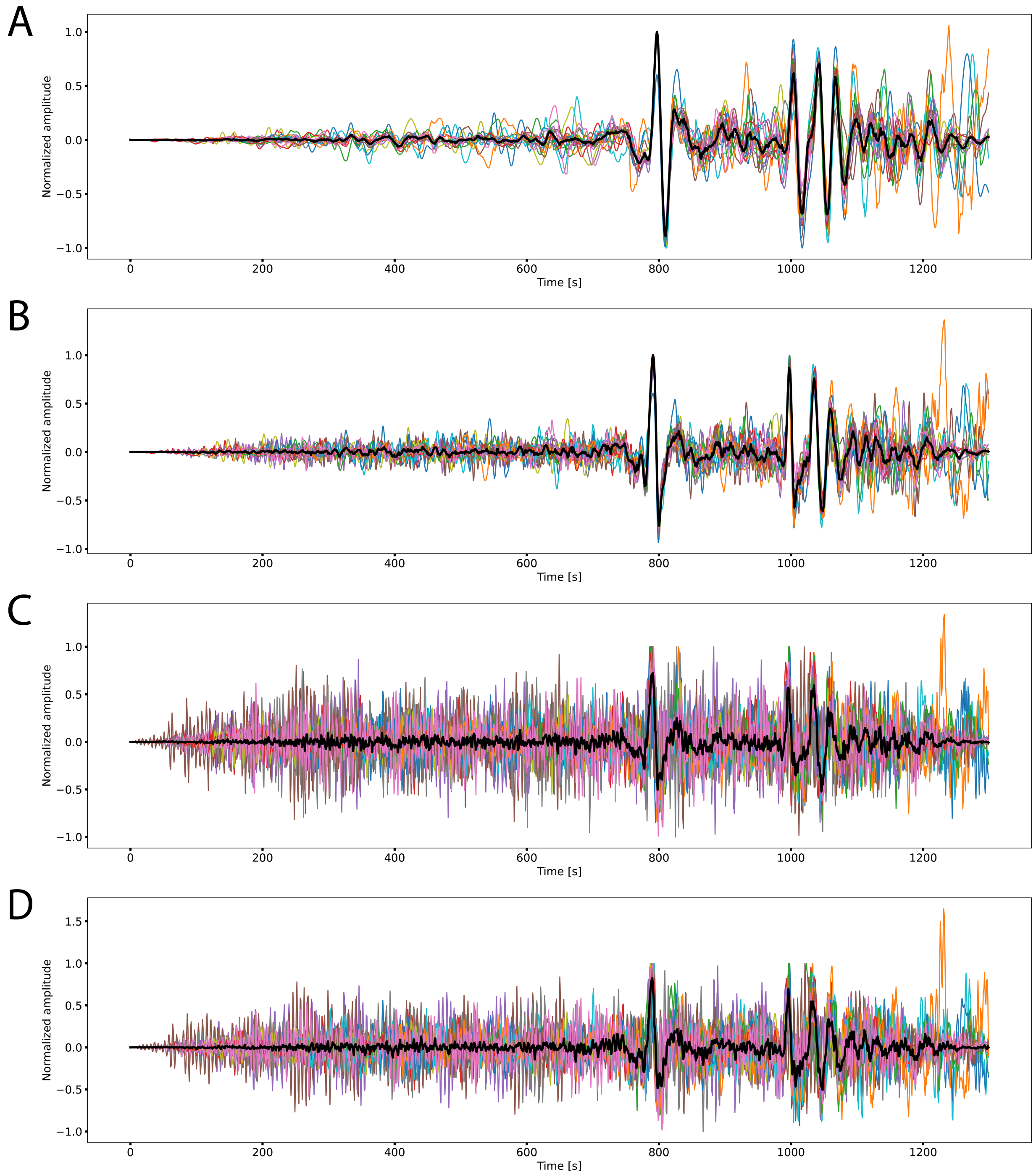

Fig. S2 – P wave ground motion stacks for different passbands. Median stack of 20° azimuthal bins stacks of P wave ground displacements normalized by the peak value around 800s for different passbands. **A:** 0.01-0.05 Hz. **B**: 0.01-0.1 Hz, **C:** 0.01-0.2 Hz, **D:** 0.01-0.4 Hz.

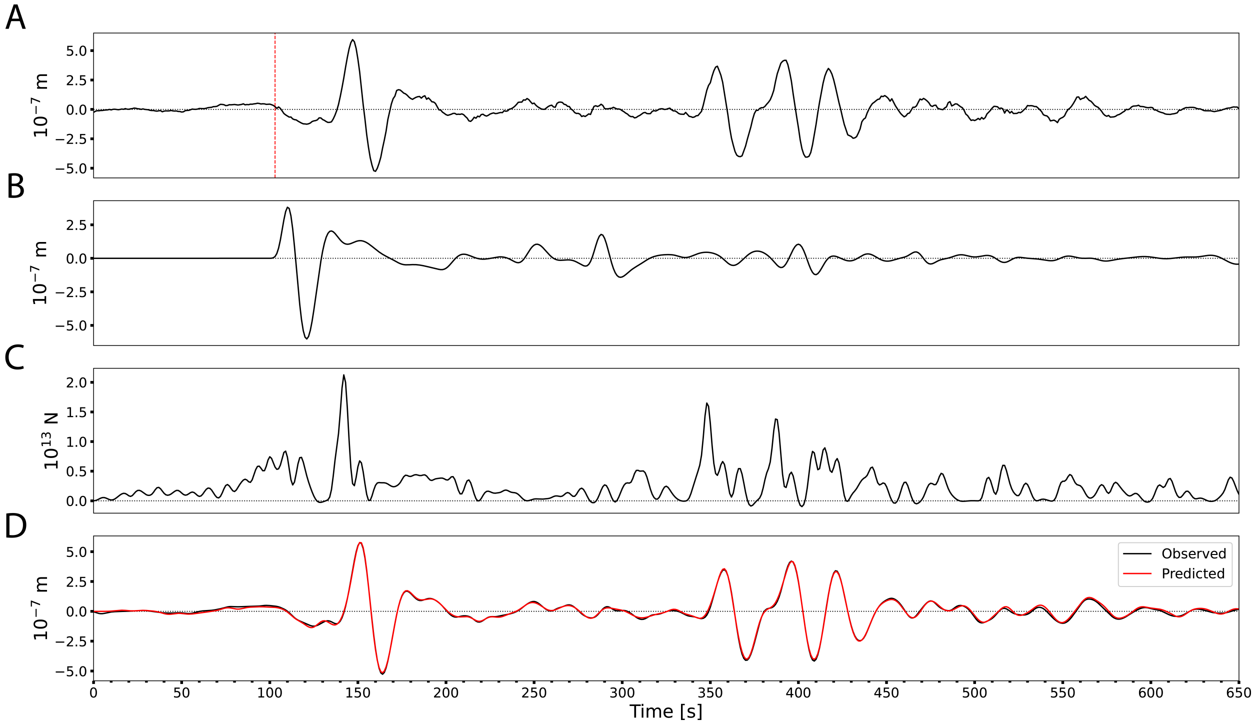

Fig. S3 – Deconvolution of the median P wave ground motion stack from Fig. 3 by a downward vertical point-force impulse response. **A**: Global median stack for 518 P wave ground displacement at 78.5° epicentral distance, causally filtered in the 0.01-0.05 Hz band. The red dotted line indicates the expected P wave arrival time for the magnitude 5.8 event reported by the U.S. Geological Survey National Earthquake Information Center, as in Fig. 3. **B:** Synthetic P wave impulse-response seismogram at 78.5° epicentral distance for a downward vertical point force with impulse strength 10^{14} N, acting on the surface of model AK135, causally filtered in the 0.01-0.05 Hz band. **C:** Force time history at the source obtained by positivity constrained iterative deconvolution of **A** and **B**. **D:** Comparison between the observed P waveform (black curve) and the reconstituted (red curve) P waveform obtained by convolving the reaction force time series (**C**) with the impulse response seismogram (**B**). **E:** Same as **A** but for model PREM_a. **F:** Force time history at the source obtained by positivity constrained iterative deconvolution of **A** and **E**. **G:** Comparison between the observed P waveform (black curve) and the reconstituted (red curve) P waveform obtained by convolving the reaction force time series (**F**) with the impulse response seismogram (**E**).

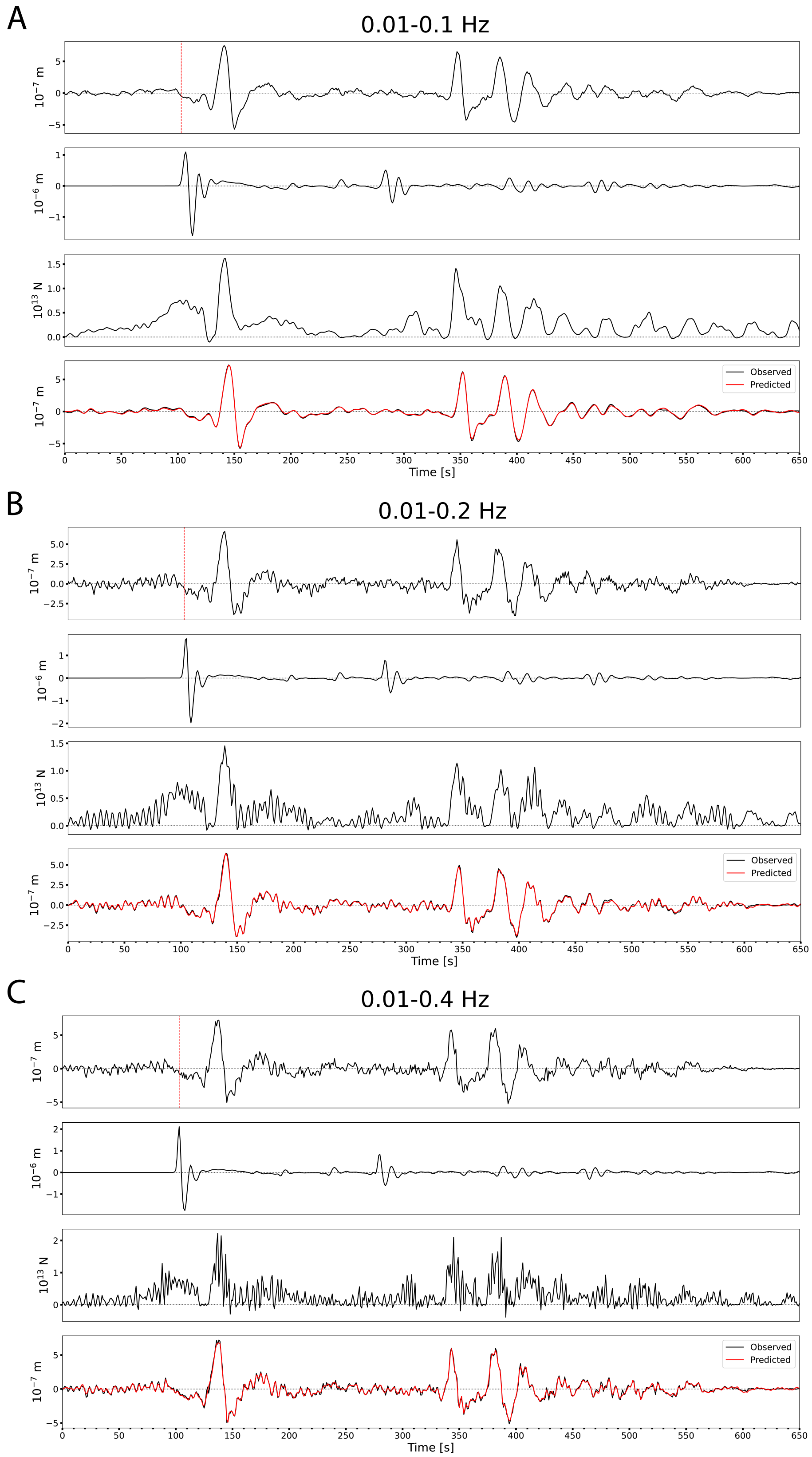

Fig. S4 – Deconvolution results for data and downward vertical point-force response synthetic seismograms for model AK135, with format similar to S3, but filtered in 3 different frequency bands. **A**: 0.01-0.1 Hz; **B**: 0.01-0.2 Hz, **C**: 0.01-0.4 Hz.

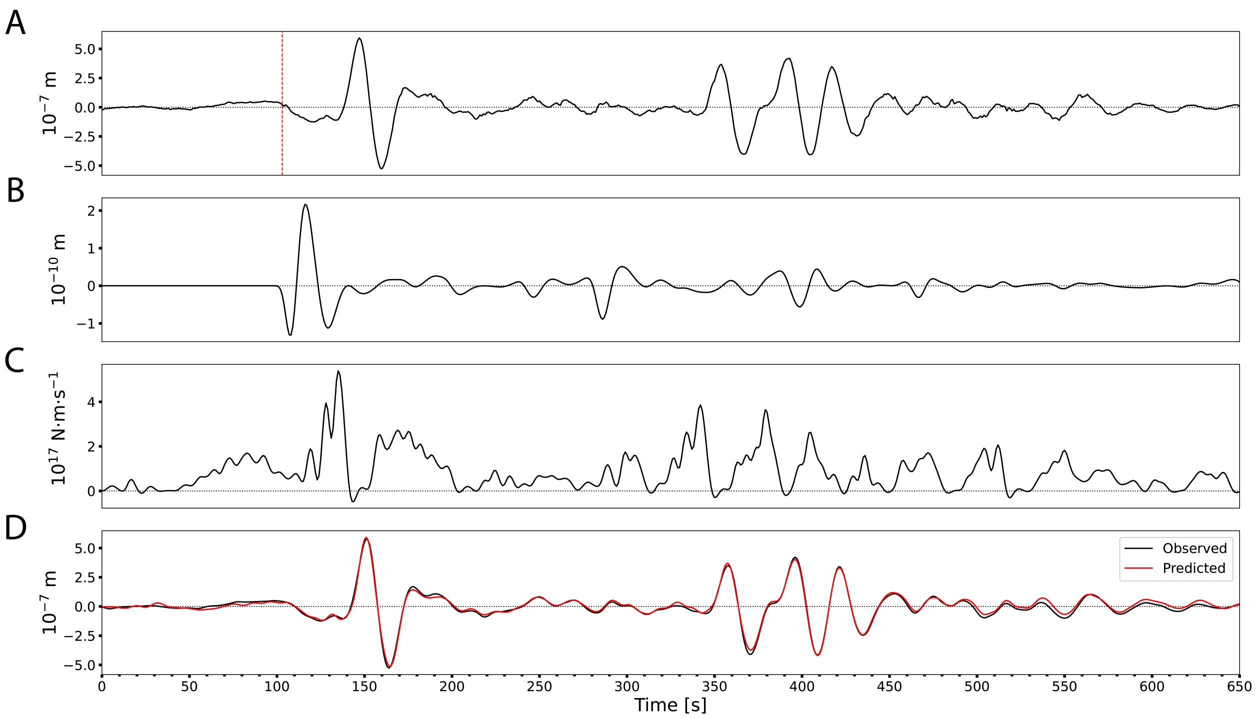

Fig. S5 – Deconvolution of the P wave stack by an implosion impulse response. A: Global stack for P wave ground displacement filtered in the 0.01-0.05 Hz band at 78.5° epicentral distance (same as Fig. S3A). The red dotted line indicates the expected P wave arrival time for the magnitude 5.8 event reported by the U.S. Geological Survey National Earthquake Information Center, as in Fig. 3. **B:** Synthetic P wave impulse response seismogram at 78.5° epicentral distance for an isotropic implosive point-source with seismic moment of 10^{15} N-m located at 5 km depth in model AK135, filtered in the 0.01-0.05 Hz passband. **C:** Moment rate source-time function obtained by iterative deconvolution with positivity constraint. **D:** Comparison between the observed P waveform (black curve) and the reconstituted waveform (red curve) obtained by convolving the implosion moment rate function time series (**C**) with the impulse response seismogram (**B**).

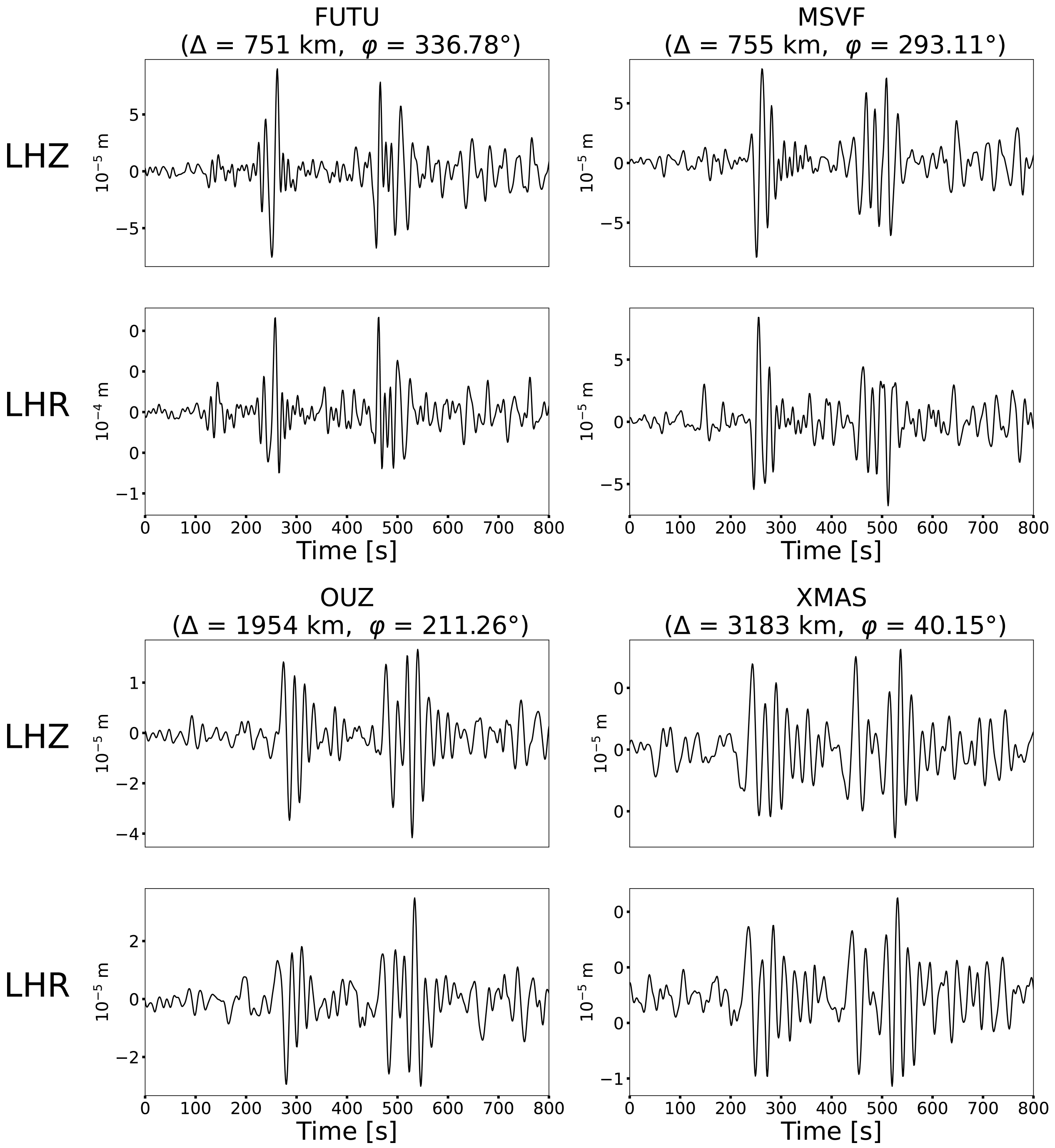

Fig. S6 – Vertical (LHZ) and radial (LHR) ground displacement time series in the passband 0.01-0.1 Hz at 4 different stations. Time is relative to the magnitude 5.8 event origin time. The wavepackets are primarily Rayleigh waves generated at the source. Δ = epicentral distance from source to each station in km. φ = azimuth from the source to each station.

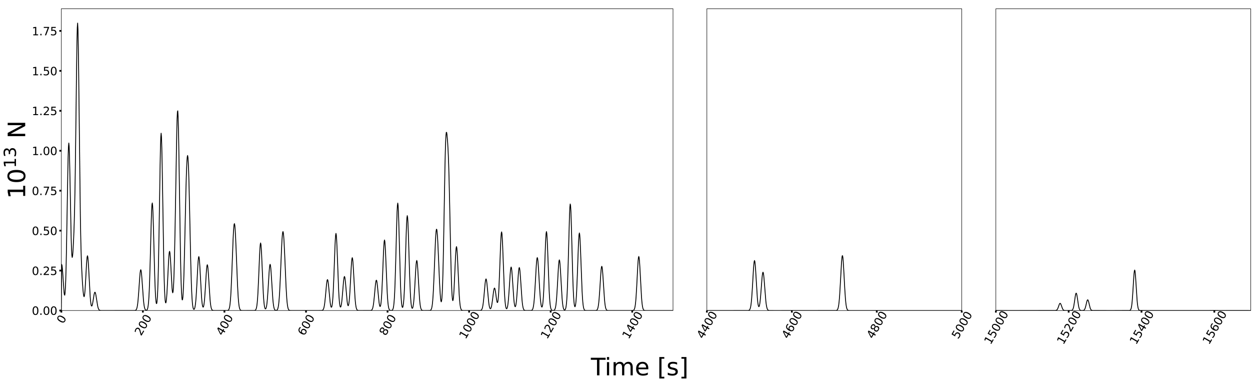

Fig. S7 – Complete time series of the force time history obtained from the simulated annealing waveform inversion. The solution was optimized by fitting the time and amplitude of pulses for windows spanning from 0 - 1500 s, 4400-5000 s and 15000-15700 s.

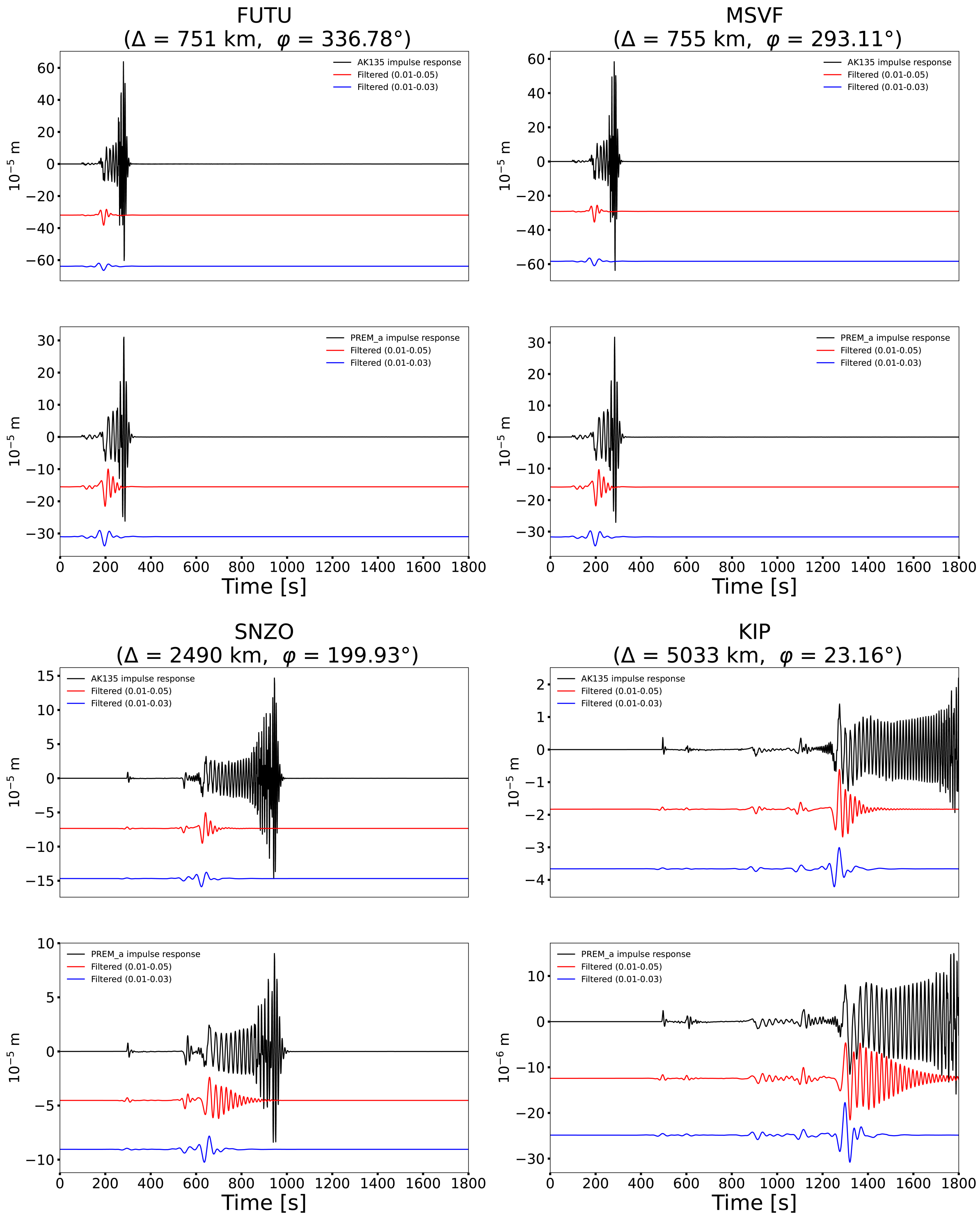

Fig. S8 – Vertical component vertical point-force responses at four stations for the 1D AK135 and PREM_a reference Earth models. The epicentral distance (*Δ*) and azimuth (*φ*) of each station is indicated below the station name. The broadband impulse displacements are shown in black, with red traces showing the 0.01-0.05 Hz bandpass filtered signals and blue traces showing the 0.01-0.03 Hz bandpass filtered signals. The strong differences in high-frequency (>0.05 Hz) Rayleigh wave dispersion for the two models are greatly reduced in the passband of the actual data, but minor differences in dispersion are still apparent.

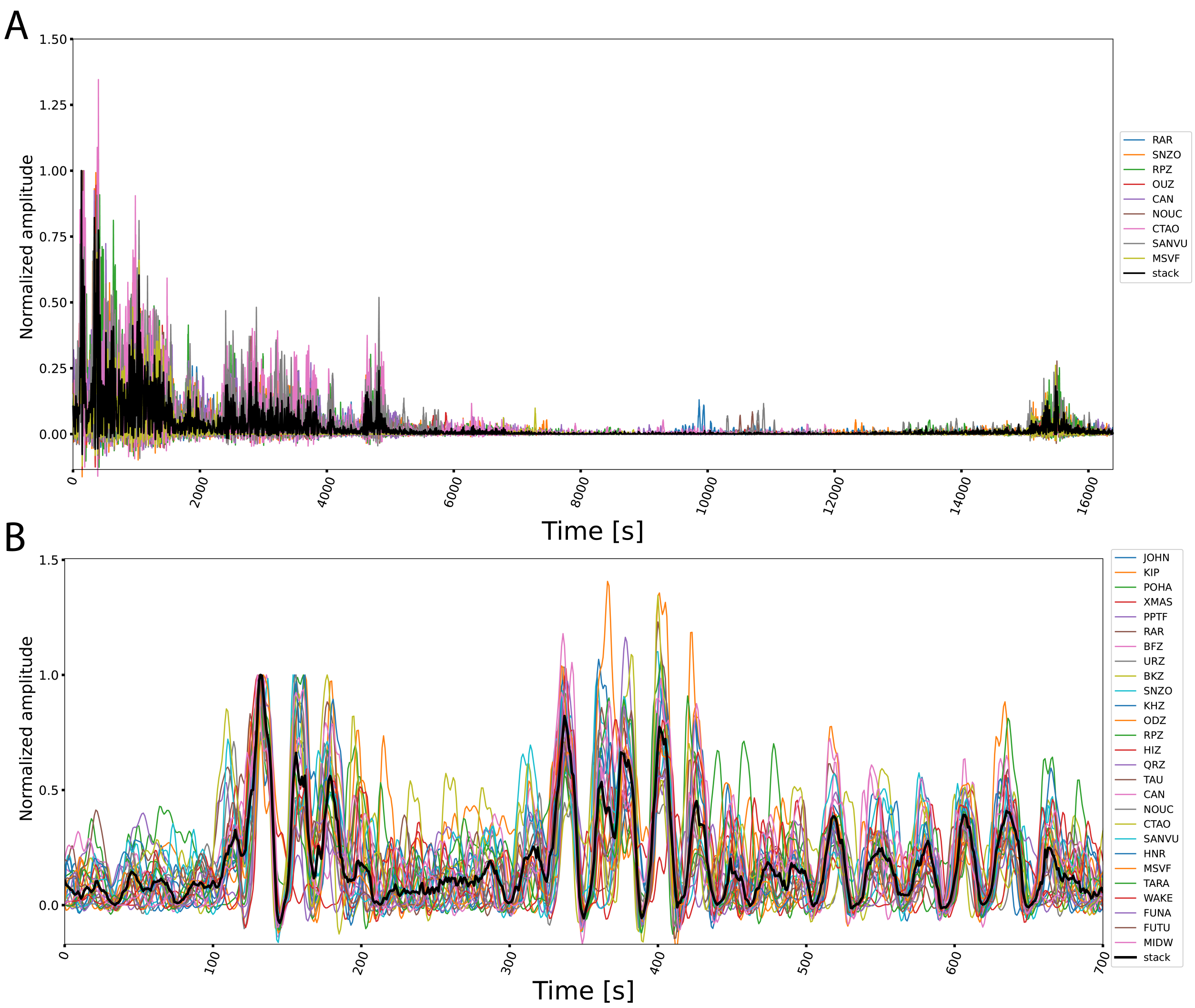

Fig. S9 – Amplitude-normalized force time histories from stacking of Rayleigh wave deconvolutions of vertical components filtered in the 0.01-0.1 Hz band for the AK135 Earth model. A: Force-time function F(t) extending through ~16,500 s of the eruptive process. Individual station deconvolutions are shown with color traces with the legend on the right. The black curve is the median stack of the aligned F(t) estimates. **B:** The initial 700s of the aligned F(t), with individual stations shown by color lines and the median stack shown by the black line. Note that different stations were screened for stacking F(t) for different time intervals due to differences in signal-to-noise ratios during the ~4.5 hr long eruption.The traces are not shifted to absolute time and the timing here depends on the relative alignment of the F(t) obtained with the AK135 model.

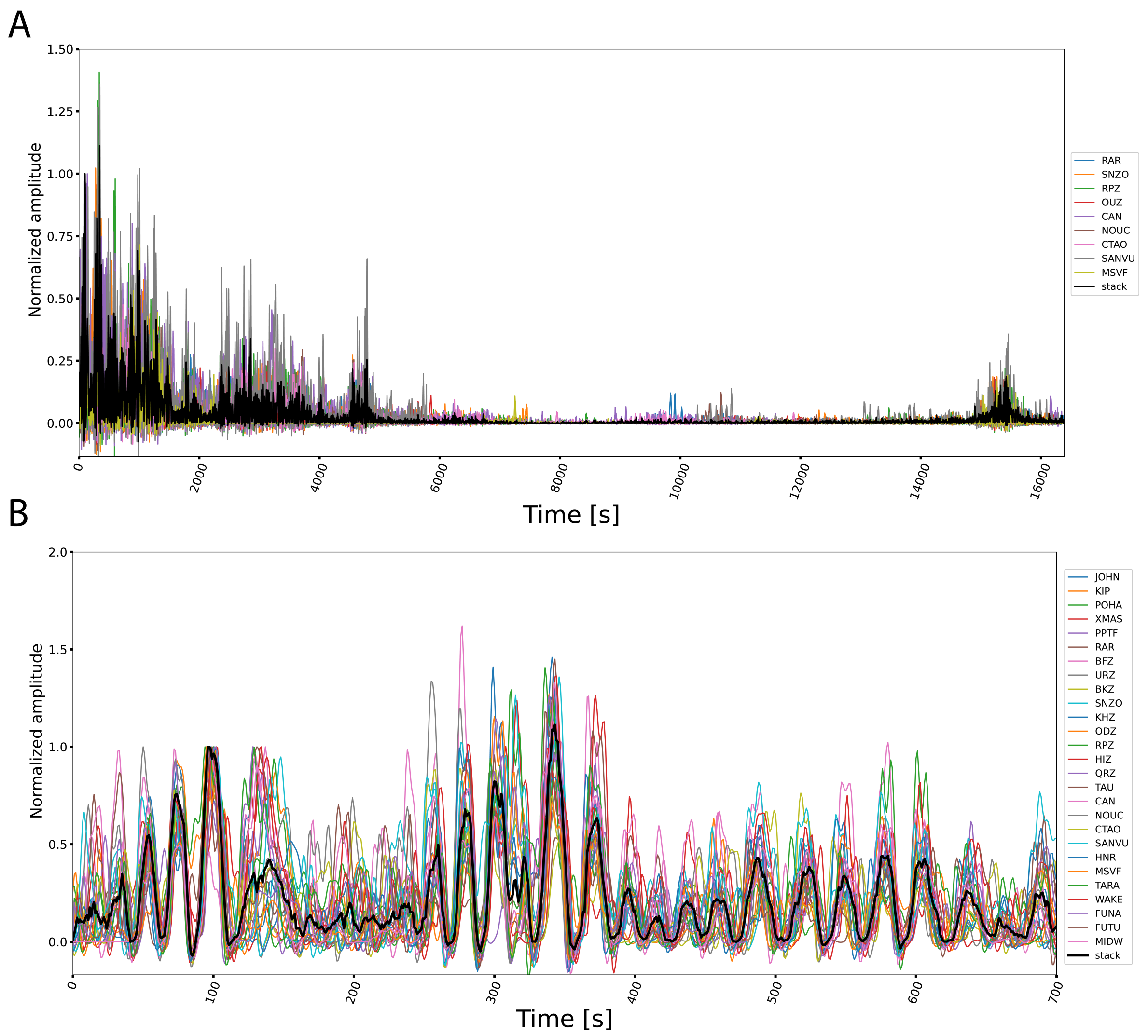

Fig. S10 – Amplitude-normalized force time histories from stacking of Rayleigh wave deconvolutions of vertical components filtered in the 0.01-0.1 Hz band for the PREM_a Earth model. A: Force-time function F(t) extending through ~16,500 s of the eruptive process. Individual station deconvolutions are shown with color traces with the legend on the right. The black curve is the median stack of the aligned F(t) estimates. **B:** The initial 700s of the aligned F(t), with individual stations shown with color lines and the median stack shown by the black line. Note that different stations were screened for stacking F(t) for different time intervals due to differences in signal-to-noise ratios during the ~4.5 hr long eruption. The PREM_a Earth model causes some side-lobes in the deconvolution due to slight errors in Rayleigh wave dispersion in the data passband, but the major features are similar to Fig. S9. The traces are not shifted to absolute time and the timing here depends on the relative alignment of the F(t) obtained with the PREM_a model.

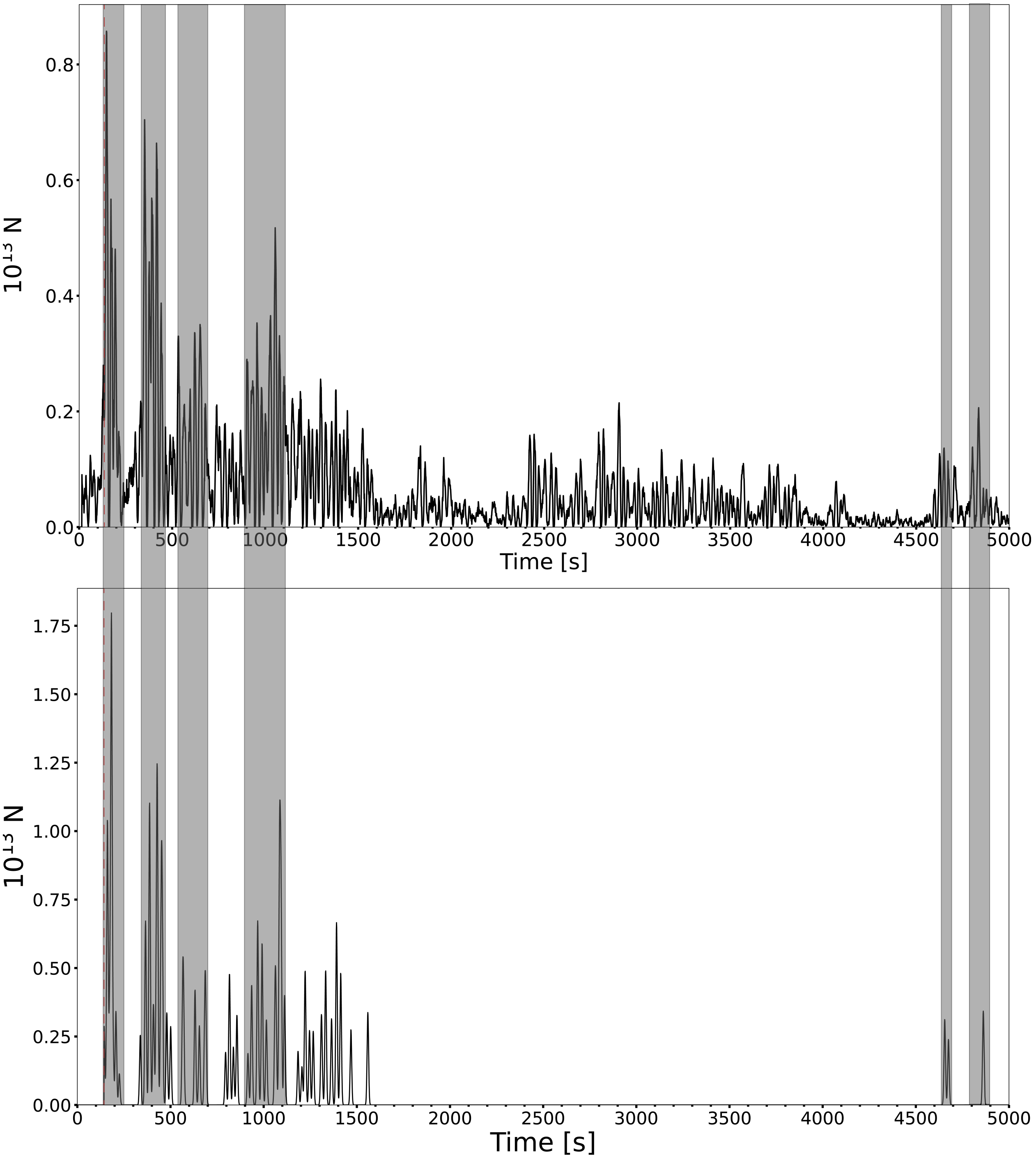

Fig. S11 – Comparison of 5000 s of the force time history calculated from the regional surface waves deconvolution (A) and direct fitting of regional surface waves by the simulated annealing inversion (B).

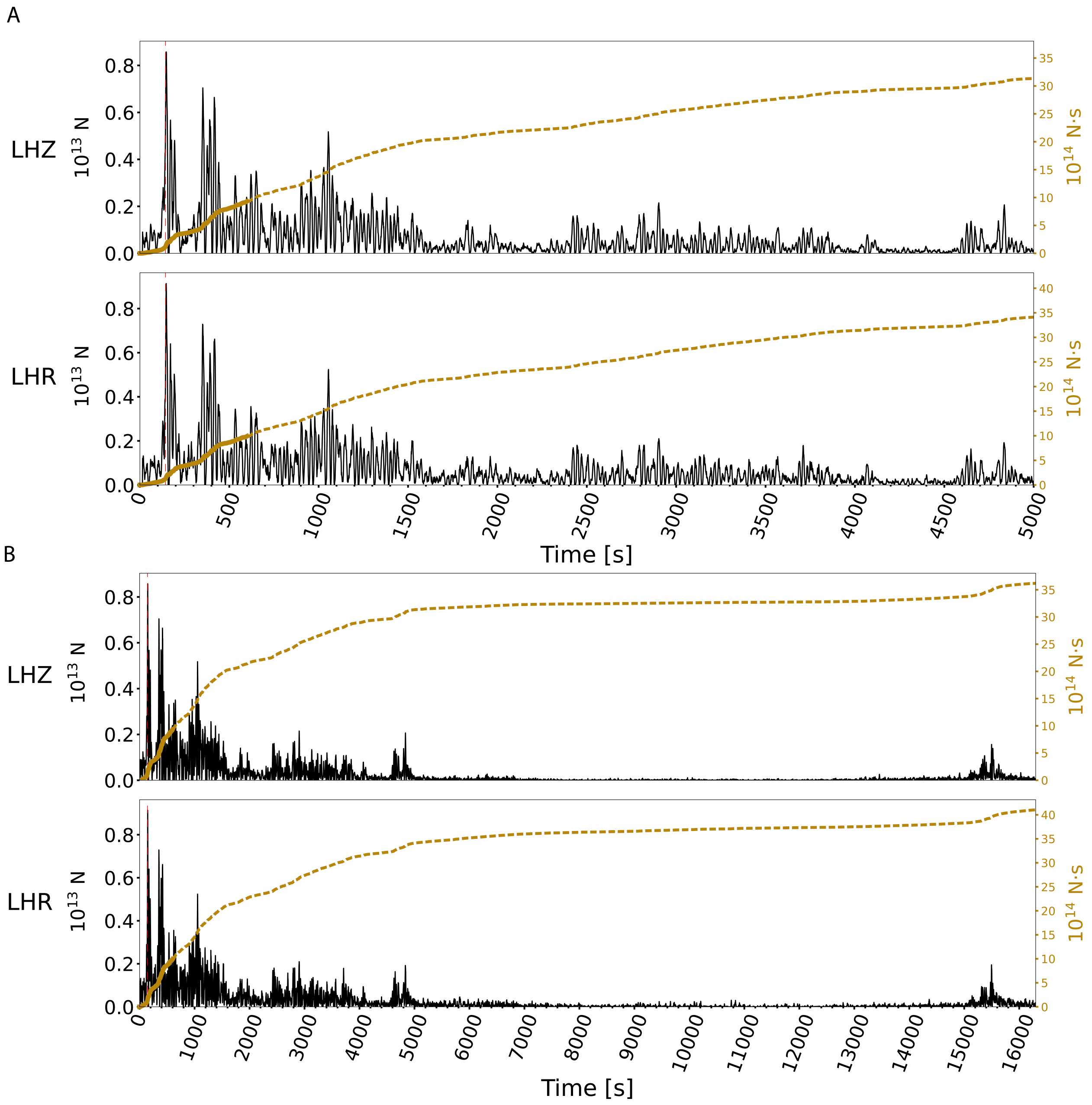

Fig. S12. Vertical reaction force (black) and force impulse (gold) time history from Rayleigh waves. The median stack vertical point-force time histories obtained by deconvolving individual displacement recordings extending for 5000 s (A) and 16,300 s (B) after the source began by corresponding Green's functions for vertical motion (LHZ) and horizontal motion (LHR) of short-arc Rayleigh waves. The uncertainty of the force impulse increases ~10 minutes after the origin time of the M5.8 event (gold dashed line), since the established (two sided) atmospheric oscillatory forces could interfere with the purely positive vertical force.
